# Cross-IgE sensitization correlates with cross-anaphylaxis among peanut and multiple tree nuts in a murine model

**DOI:** 10.3389/falgy.2025.1720870

**Published:** 2026-02-10

**Authors:** Anish Maskey, Michelle Carnazza, Daniel Kopulos, Madison Spears, Nan Yang, Raj K. Tiwari, Jan Geliebter, Soheila Maleki, Xiu-Min Li

**Affiliations:** 1 Department of Pathology, Microbiology & Immunology, New York Medical College, Valhalla, NY, United States; 2 Department of Microbiology & Immunology, Touro College of Osteopathic Medicine, Great Falls, MT, United States; 3 Division of R&D, General Nutraceutical Technology, LLC, Briarcliff Manor, NY, United States; 4 Department of Otolaryngology, New York Medical College, Valhalla, NY, United States; 5 United States Department of Agriculture, Agriculture Research Service, Southern Regional Research Center, New Orleans, LA, United States; 6 Department of Dermatology, New York Medical College, Valhalla, NY, United States

**Keywords:** correlation analysis, cross-reactive anaphylaxis, cross-reactive IgE, multiple food allergy, PNA/TNA

## Abstract

**Background:**

The incidence of multiple food allergies (MFA), defined as exhibiting allergic responses to two or more distinct food groups, has been increasing. Since peanut (PN)/ tree nuts (TN) MFA causes severe reactions, it is challenging to study cross-reactivity immediately using human subjects. Development of a PN/TN cross-reactivity model may provide a useful tool to understand the immunological mechanisms underlying cross-reactivity among PN/TN, and a tool to develop therapies that prevent cross-reactivity.

**Methods:**

Sensitization to the most common allergens, PN, walnut (WN), and cashew (CSH), were utilized for cross-reactive sensitization to eight other TNs (almond, pecan, pistachio, hazelnut, Brazil nut, pine nut, macadamia but, and coconut). C3H/ HeJ mice were intraperitoneally sensitized (primed) with a mixture of PN, WN, and CSH and specific (s)- IgE levels against the primed and cross-reactive TN antigens were determined. Intragastric challenges with each primed and eight unprimed allergens were performed and anaphylaxis symptoms measured. Correlation of primed IgE levels and symptoms scores with primed and unprimed allergen were conducted.

**Results:**

PN, WN, and CSH sensitization induced significant cross reactivity against other TNs, with elevated sIgE levels against both primed and unprimed allergens. Cross-reactivity was confirmed clinically, with anaphylaxis upon primed and unprimed nut challenges, exhibiting strong positive correlations among sIgE levels and anaphylaxis observed.

**Conclusion:**

Interestingly, as seen with patients, different priming nuts were capable of cross-sensitization against different groups of challenge nuts. Thus, we present a model system that can be developed to investigate the molecular basis of MFA and potential therapeutic approaches.

## 1 Introduction

Food allergy (FA) is a growing health concern as its incidence has increased dramatically in Westernized countries over the last few decades. Burdening approximately 32 million people in the United States (US), FA affects an estimated 8% of children and 6% of adults (1). While there have been 170 foods reported to cause an allergic reaction, only 8–9 are responsible for 90% of allergies. Milk, egg, wheat, and soy constitute 85% of childhood food allergies and are largely grown out by 5 years of age (2–4). However, persistent food allergy is seen with sensitivity to peanuts (PNA), tree nuts (TNA), fish, shellfish, and sesame as only 2%–20% of patients will outgrow these allergies by adulthood (5, 6).

The first exposure to an ingested allergen is typically asymptomatic, however results in the production of an IgE-dependent hypersensitivity response, primarily generated by IgE-producing plasma cells. The Fc portion of the secreted IgE binds its cognate receptor on innate immune cells, including mast cells, resulting in their sensitization. With subsequent exposure, the allergen stimulates these IgE-coated cells to degranulate resulting in an allergic reaction (7). Organ symptoms of allergic reactions depend on where the degranulation of histamine and other inflammatory mediators including leukotrienes and prostaglandins by the sensitized cells occurs, how much antigen exposure occurs, and how sensitive the individual is. These can be mild symptoms such as tingling or itching mouth, hives, swelling, wheezing, vomiting, diarrhea, abdominal pain. In severe and systemic cases, anaphylaxis can occur if the allergens enters circulation. Over 40% of children and 50% of adults with food allergies have experienced this type of reaction (8).

Alarmingly, persistent PNA and TNA account for most of the fatal and near-fatal anaphylactic reactions (9, 10). Of the tree nuts, the most common allergies include walnuts, cashew, hazelnut, and pistachio. Individuals with allergies to a single food may avoid other foods because of the history of reaction, positive tests without prior ingestion or reaction, or concerns over shared allergens. Studies indicate that in the US, 40% of children and 48% of adults with food allergies have multiple food allergies (MFA) (11). Diagnosis of MFA does not simply mean “more allergies”, but is a complex condition characterized by distinct biological mechanisms constituting a higher burden, greater avoidance, increased risk, and more complex management than a single FA (12). Interestingly, Maloney et al. showed that in 324 PNA patients, 86% were also sensitized to tree nuts and 34% had clinically documented allergy (13). In a study conducted by Andorf et al. (14), of 55 children in the US, TNA is associated with a 54%–60% incidence of PNA, with no single TNA being more likely associated with confirmed PNA than another. A population-based study of MFA epidemiology aiming to identify specific co-allergen clustering in both pediatric and adult subpopulations confirmed this, identifying peanut/tree-nut dominant MFA affecting 28% of children and 17% of adults in the US (11). It has been observed that these MFA sufferers have an increased rate and severity of food allergic reactions and emergency room visits compared to a single nut allergy (11). The exacerbation of these outcomes in people with PNA/TNA MFA urges the need for investigation and therapeutics that could alleviate the burden on FA sufferers and their families. Antihistamines and glucocorticoids do not prevent the progression of an allergic reaction, nor do they treat anaphylaxis as epinephrine does. Aside from avoidance and rescue medication, treatment and preventive interventions including biologics and oral immunotherapies (OIT), are underwhelming. Bortezomib, a proteosomal inhibitor that depletes plasma cells, is associated with substantial toxicity and broad immunosuppression, limiting its use for FA (15–17). Omalizumb (Xolair), an anti-IgE antibody, “traps” IgE but does not halt its production and therefore does not provide long-term protection (18). Palforzia, an OIT designed to build peanut tolerance, only, does not decrease the production of IgE, but paradoxically increases IgE levels, increasing the risk for immune reactions (19–21). Therefore, instead of subjecting individuals to risk via oral food challenges, we can utilize mouse models for studying cross-reactivity *in vivo* for this prevalent multi-food allergy group.

Immunological reactivity is thought to be due to IgE responses to shared allergens or similar allergenic molecules. Several common families of protein allergens have been identified in these foods, including 2S albumins (22). For example, major peanut allergen, Ara h 2, also has been shown to share common IgE-binding epitopes with walnut (23), almond and Brazil nut allergens. Consequently, the binding of IgE to these allergens contributes to allergic reactivity and potential cross-reactivity. *In vivo*, cashew and walnut T cell epitope homology has been demonstrated (24). Further, peanut specific IgE antibodies of two peanut-allergic patients demonstrated cross-reactivity with tree nut allergens causing basophil activation *in vitro* (25). It is speculated that the affinity of the IgE-epitope interaction dictates clinically relevant cross-reactivity (26).

Studies on MFA have shown that in a subset of children with positive skin prick tests to several foods, only one-third of that correlated with positive food challenges and that specific IgE levels to food does not imply true food allergy (27). We have previously demonstrated a comprehensive murine model of IgE-mediated multiple food groups of cross-reactive anaphylaxes across several tree nuts (28). In another study, walnut and pecan allergy demonstrated correlations with IgE levels and severity of anaphylaxis *in vivo* (29). There is a lack of sufficient and standardized preclinical evidence that is reproducible for clinically meaningful multi-nut sensitization that can robustly elucidate therapeutic and prophylactic efficacy in humans. Models of cross-sensitization have yet to be well- established that include findings beyond serology. There is a critical need for preclinical models capable of discerning therapeutic and prophylactic strategies that appear effective in single-allergen systems that ultimately fail to translate to humans. Our research therefore aimed to establish a PN/TN MFA model to extend previous findings to a larger group of tree nuts following sensitization with highly prevalent food allergens, PN, CSH, and WN and establish the correlation between cross-reactive IgE and cross-reactive anaphylaxis symptom scores. The development of an established model can then be used as a reliable and reproducible preclinical evaluation of therapeutics and prophylactics for MFA.

## 2 Materials & methods

### 2.1 Crude nut extract preparation

For sensitization, crude nut (PF Snacks, Richmond, VA) was extracted from defatted powder by standard acetone extraction (PN, WN, CSH). Briefly, 200 mg of PN powder was mixed in PBS and vortexed until dissolved. This was centrifuged and filtered. Protein concentration was assessed by Bradford assay (Table 1). The same method was utilized for WN and CSH. 500 μg each of PN, CSH, and WN extract were used for sensitization.

**Table 1:** Concentration of allergens.

Primed allergen		Cross-Reactive allergen	
Protein	Concentration (mg/mL)	Protein	Concentration (mg/mL)
Peanut	27.1	Almond	69.7
Walnut	33.0	Pecan	96.4
Cashew	40.2	Pistachio	49.3
		Hazelnut	28.5
		Brazil nut	54.9
		Pine nut	59.8
		Macadamia nut	25.0
		Coconut	7.0

Protein concentration of each allergen that was assessed by Bradford assay and used for sensitization and/or challenge protocols.

For challenge, 20 g of the crude nut (PF Snacks) soaked in 100 mL PBS for 20 min and then blended periodically over 3 h. Following fine nut suspension (200 mg/mL), 16.5 μL/mL vodka (Stolichnaya) and 1.5% (w/v) sodium bicarbonate were added freshly prepared. 500 μL of this feeding solution was used for challenge. Crude nuts for challenge included PN, WN, CSH (primed nut allergens) almond (ALM), pecan (PCN), pistachio (PSC), hazelnut (HZN), Brazil nut (BZN), pine nut (PINE), macadamia nut (MCDM), and coconut (COCO) (cross reactive allergens) (Table 1).

### 2.2 Mice

C3H/HeJ mice (female, 8 weeks old) were obtained from Jackson Laboratory (Bar Harbor, ME). These mice are susceptible to oral anaphylaxis, making them ideal for these studies (30). Mice were kept in a specific pathogen-free environment and fed allergen-free chow, following standard animal care guidelines. All animal experiments conducted in this study were approved and carried out in strict compliance with the regulations and protocols set forth by the Institutional Animal Care and Use Committee of New York Medical College (Protocol Number 15156).

### 2.3 Peanut, cashew and walnut allergen sensitization (priming), boosting, and challenge

**Figure 1:**
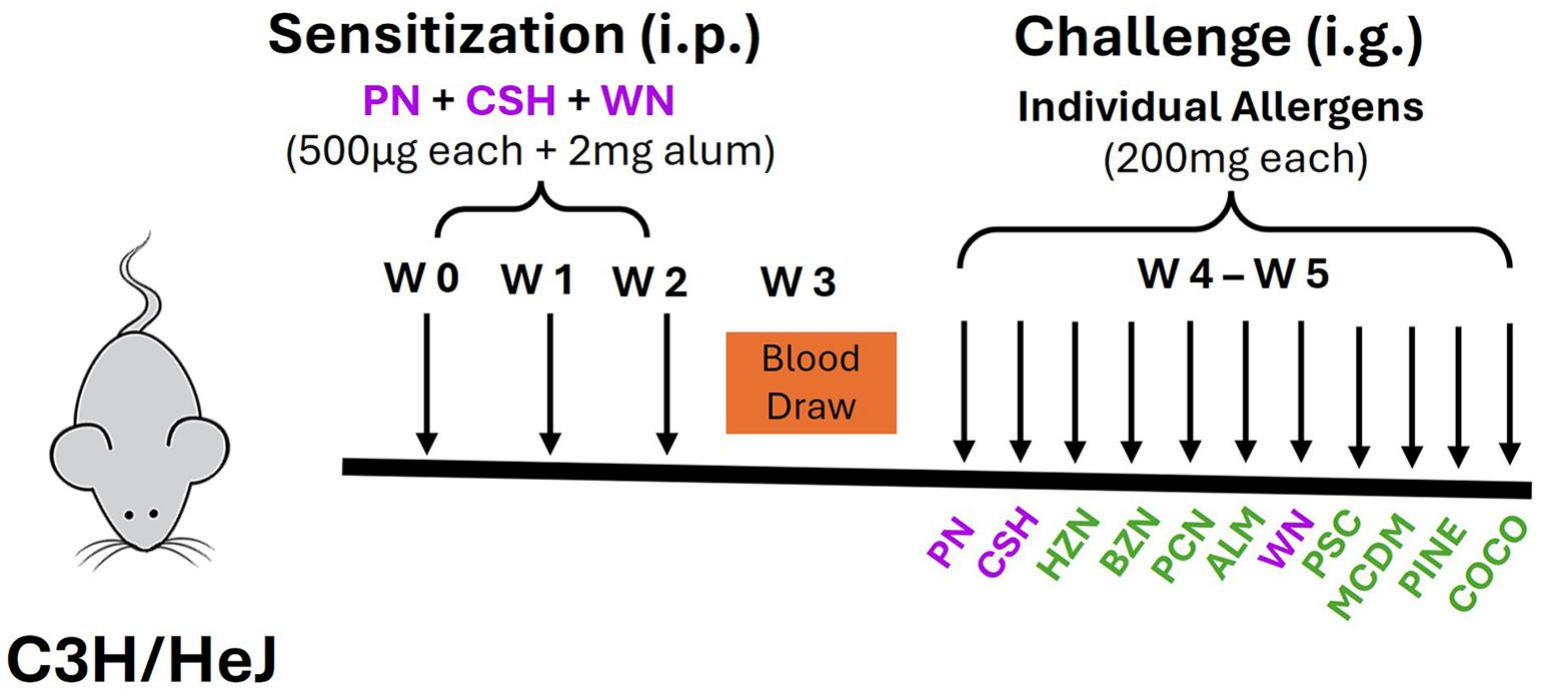
Multi-nut cross-sensitization protocol. C3H/HeJ mice (*n* = 24) were sensitized intraperitoneally (i.p.) with 500 μg of peanut, cashew, and walnut (PN/CSH/WN) crude extract adsorbed in 2 mg alum weekly for 3 weeks. Blood was drawn at week 3 to determine cross-sensitization between allergens vs. Naïve. Mice were challenged over 10-day period and anaphylaxis scores were evaluated. ALM, almond; BZN, Brazil nut; CSH, cashew; CO, coconut; HZN, hazelnut; MCDM, macadamia nut; PCN, pecan; PINE, pine nut; PN, peanut; PSC, pistachio; WN, walnut.

C3H/HeJ mice (*n* = 24) were sensitized (primed) intraperitoneally (i.p.) with 500 μg each of PN/CSH/WN crude extract adsorbed in 2 mg alum (10 mg/mL, G Biosciences, St. Louis, MO) in 200 μL PBS, weekly for 3 weeks (Figure 1). Blood was drawn at week 4 to determine allergen-specific IgE sensitization to primed antigens (PN, CSH, WN) and cross-sensitized (unprimed) TN antigens (ALM, PCN, PSC, HZN, BZN, MCDM, PINE, and COCO) vs. naïve mice. From week 5, mice received daily intragastric (i.g) challenges of one nut each day at 200 mg/mouse sequentially, beginning with primed antigens PN, CSH, and WN followed by unprimed antigens (ALM, PCN, PSC, HZN, BZN, MCDM, PINE, and COCO) in total 11 nuts over 11 days. Rectal temperatures and anaphylaxis symptom scores were recorded every 10 min for 90 min.

### 2.4 Measurement of allergen-specific IgE levels

Blood was collected by the submandibular vein and stored at −80 °C for analysis. IgE levels were assessed by coating ELISA plates with crude protein extracts (500μg/mL) in coating buffer of primed antigens (PN, CSH, WN), and cross reactive antigens (unprimed antigens: ALM, PCN, PSC, HZN, BZN, MCDM, PINE, and COCO), as well as reference control anti-mouse IgE (Pharmigen, San Diego, CA; 0.5 mg/mL), and incubated overnight at 4 °C. Plates were then washed and blocked in incubation buffer, after which serum samples were added as well as reference solution (Pharmigen, San Diego, CA; 0.5 mg/mL) in the anti-IgE-coated wells. Following overnight incubation at 4 °C, plates were washed and then underwent incubations in biotinylated detector antibody, (Pharmigen, San Diego, CA; 0.5 mg/mL) then avidin-peroxidase conjugate (Sigma-Aldrich, Saint Louis, MO), followed by ABTS (2,2′-azino-bis (3-ethylbenzothiazoline-6-sulfonic acid) (ThermoFisher, Waltham, MA). Following color development, absorbance was taken at 405 nm (28, 31).

### 2.5 Assessment of anaphylactic reactions

Following each challenge, anaphylaxis reactions were assessed every 10 min. The anaphylactic symptom scores were assigned based on the severity of symptoms as follows: 0: No signs, (1) scratching and rubbing around the nose and head, (2) puffiness and redness around the eyes and mouth, diarrhea, piloerection, reduced activity, and/or increased respiratory rate, (3) wheezing, labored respiration, and cyanosis around the mouth and tail, (4) symptoms consistent with score 3, accompanied by no activity after prodding, tremors, or convulsions, (5) death (32). The sum of all symptom scores was determined per group. Peak reaction was determined to be 40 min and used for correlation analysis.

### 2.6 Correlation analysis & statistics

All statistical analyses were performed using GraphPad Prism (Version 9, GraphPad Software, Inc, San Diego, CA). To assess the difference between primed and unprimed cross-reactive nut specific IgE in sensitized vs. naïve, we used a Mann Whitney *U* test and a *p*-value of ≤0.05 was considered statistically significant. For correlation analysis Spearman correlation analysis yielded *r*-values, whereby in our studies, *r* > 0.9 is very strongly positively correlated, *r* = 0.70–0.89 is strongly positively correlated, *r* = 0.4–0.69 is moderately positively correlated, *r* = 0.10–0.39 is weakly positively correlated, and *r* < 0.1 is a negligible correlation (33).

## 3 Results

### 3.1 Induction of specific IgE production against primed nut allergens following sensitization and eight cross-reactive tree nut allergens

**Figure 2:**
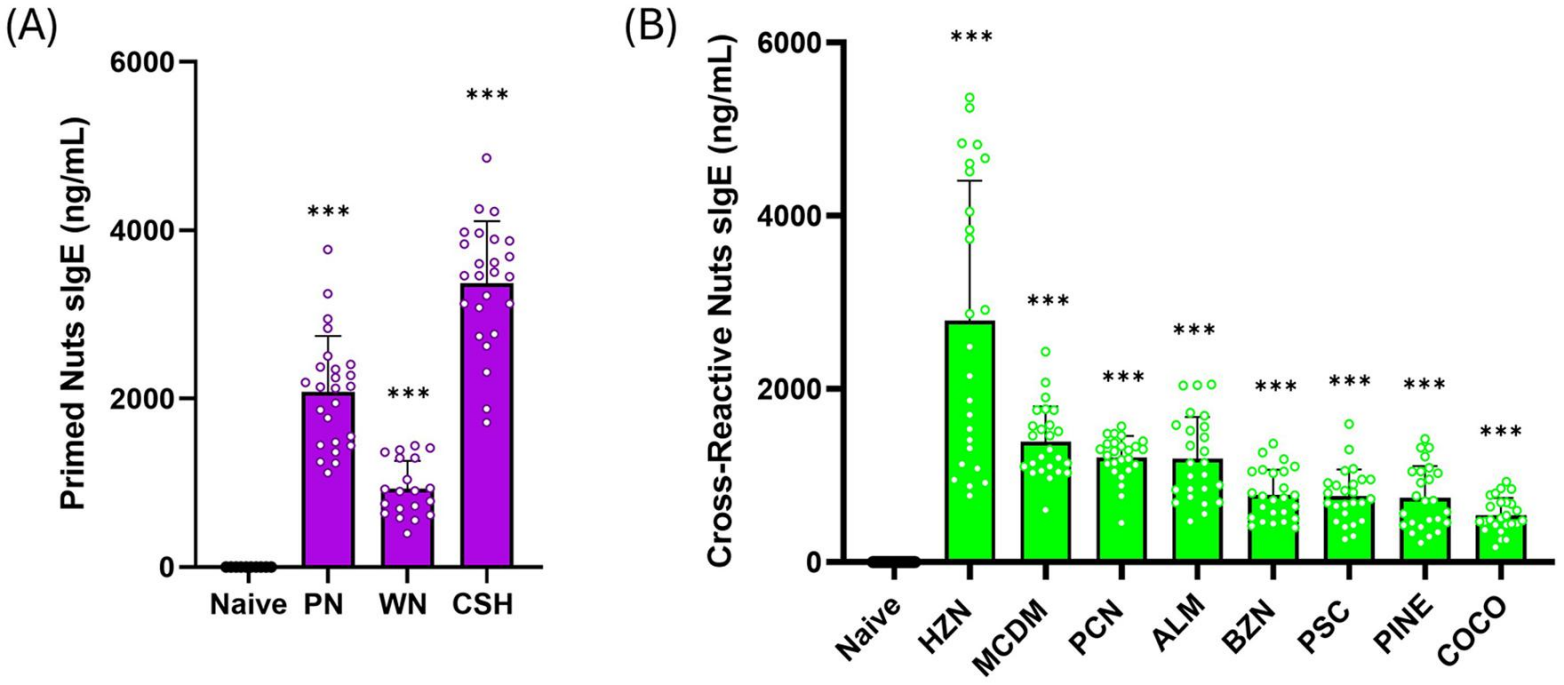
The effect of PN, WN, and CSH sensitization on primed and eight unprimed tree nut allergen IgE levels. **(A)** C3H/HeJ mice (*n*= 24) were sensitized intraperitoneally (i.p.) with 500 μg of peanut, cashew, and walnut (PN/CSH/WN) crude extract adsorbed in 2 mg alum weekly for 3 weeks. Blood was drawn at week 3 to determine IgE levels of each primed allergen in sensitized vs naïve mice. Allergen-specific IgE levels were measured by ELISA (samples were 1:20 dilution). **(B)** Cross-sensitization between other tree nut allergens in sensitized vs. naïve mice was also determined by ELISA of each individual allergen. Purple bars are primed allergens, and green bars are cross-reactive allergens. *p < 0.05, **p < 0.01; ***p < 0.001. ALM, almond; BZN, Brazil nut; CSH, cashew; COCO, coconut; HZN, hazelnut; MCDM, macadamia nut; PCN, pecan; PINE, pine nut; PN, peanut; PSC, pistachio; WN, walnut.

To determine if serum IgE produced against primed allergens would cross-react with the eight other tree nut allergens, ELISA plates were coated with the individual crude protein extract, and serum of sensitized mice were added. Mice that were sensitized to the PN, CSH, and WN mixture demonstrated high levels of sIgE to those nuts. PN IgE levels increased to an average of 2,082 ng/mL, CSH IgE levels increased to 933 ng/mL, and WN IgE averaged at 3,372 ng/mL (Figure 2A, *p* < 0.001 vs. Naive). Next we determined the levels of IgE to the unprimed eight clinically common tree nut allergens and we found they were all significantly elevated as demonstrated by the sIgE levels of HZN (2,786 ng/mL), MCDM (1,390 ng/mL) PCN (1,208 ng/mL), ALM (1,193 ng/mL), BZN (776 ng/mL), PSC (763 ng/mL), PINE (745 ng/mL) and COCO (543 ng/mL)(Figure 2B, *p* < 0.001 vs. Naive). These data demonstrated that despite not being exposed to any of the eight other tree nut allergens, mice were serologically cross- sensitized as evident by elevated sIgE levels, upon PN, WN, and CSH sensitization.

### 3.2 Challenge with primed nut allergens and cross-reactive tree nut allergens induction of anaphylactic reactions

**Figure 3:**
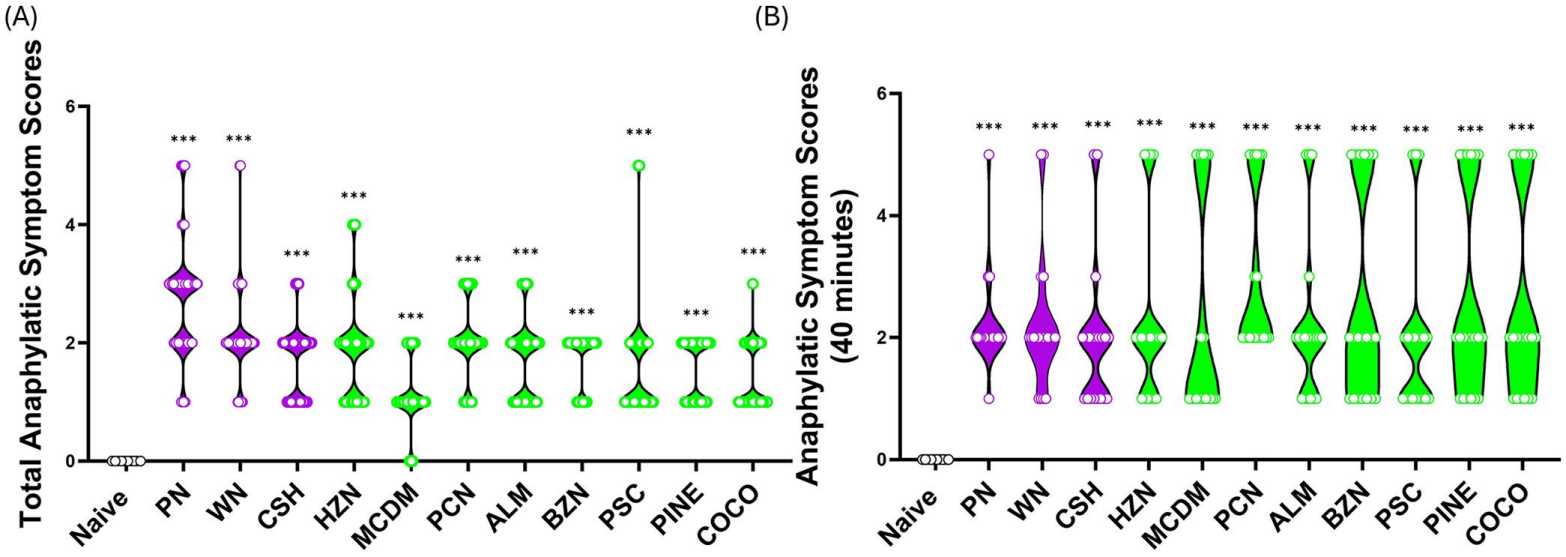
Induction of anaphylaxis with primed and cross-reactive tree nut allergens. **(A)** C3H/HeJ mice (*n* = 24) were sensitized intraperitoneally (i.p.) with 500 μg of peanut, cashew, and walnut (PN/CSH/WN) crude extract adsorbed in 2 mg alum weekly for 3 weeks, then at week 4, mice were challenged over 10-day period and symptom scores were evaluated and combined over 90 min. **(B)** Data represents the peak reaction scores recorded at 40 min post-challenge. Purple bars are primed allergens, and green bars are cross-reactive allergens. *p < 0.05, **p < 0.01; ***p < 0.001. PN, peanut; WN, walnut; CSH: cashew; HZN, hazelnut; MCDM, macadamia nut; PCN, pecan; ALM, almond; BZN, Brazil nut; PSC, pistachio; PINE, pine nut; COCO, coconut.

Following established sensitization and cross-sensitization evident by elevating sIgE levels to individual nuts, intragastric (i.g.) oral food challenges were conducted using 200 mg of freshly prepared PN, WN, and CSH (primed allergens), as well as ALM, PCN, PSC, HZN, BZN, MCDM, PINE, and COCO (unprimed allergens). Mice that were sensitized to PN, WN, and CSH showed significantly higher anaphylactic symptom scores when assessed every 10 min for 90 min upon challenge with PN (2.7 ± 0.06), WN (2.1 ± 0.04), or CSH (1.7 ± 0.04) compared to unsensitized naïve mice (Figure 3A, *p* < 0.001). Additionally, mice sensitized to PN, WN, and CSH showed higher total anaphylaxis symptom scores over 90 min when challenged with HZN (1.8 ± 0.05), MCDM (1.1 ± 0.04), PCN (2.0 ± 0.05), ALM (1.7 ± 0.05), BZN (1.7 ± 0.04), PSC (1.5 ± 0.05), PINE (1.6 ± 0.03), and COCO (1.4 ± 0.04), despite not being previously exposed to these allergens (Figure 3A, *p* < 0.001). Peak anaphylaxis was apparent at 40 min, following the second challenge. These mice also had higher peak anaphylactic symptom scores at 40 min, when challenged with PN (2.2 ± 0.17), WN (2.2 ± 0.24), or CSH (2.0 ± 0.27) compared to naïve mice that were not sensitized to any allergens (Figure 3B, ***p < 0.001). Further, the same was seen as significantly higher peak anaphylactic symptom scores at 40 min were observed when challenged with unprimed tree nuts HZN (2.3 ± 0.32), MCDM (2.3 ± 0.41), PCN (3.0 ± 0.30), ALM (2.2 ± 0.30), BZN (2.6 ± 0.38), PSC (2.0 ± 0.31), PINE (2.6 ± 0.37), and COCO (2.6 ± 0.37) (Figure 3B, ***p < 0.001). These data demonstrate that priming mice with PN, CSH, and WN is sufficient to induce anaphylaxis to the primed allergens, as well as to the unprimed tree nut allergens, supporting the occurrence of cross-anaphylaxis in this model.

### 3.3 Correlation of individual nut allergens IgE and anaphylaxis symptom scores

**Figure 4:**
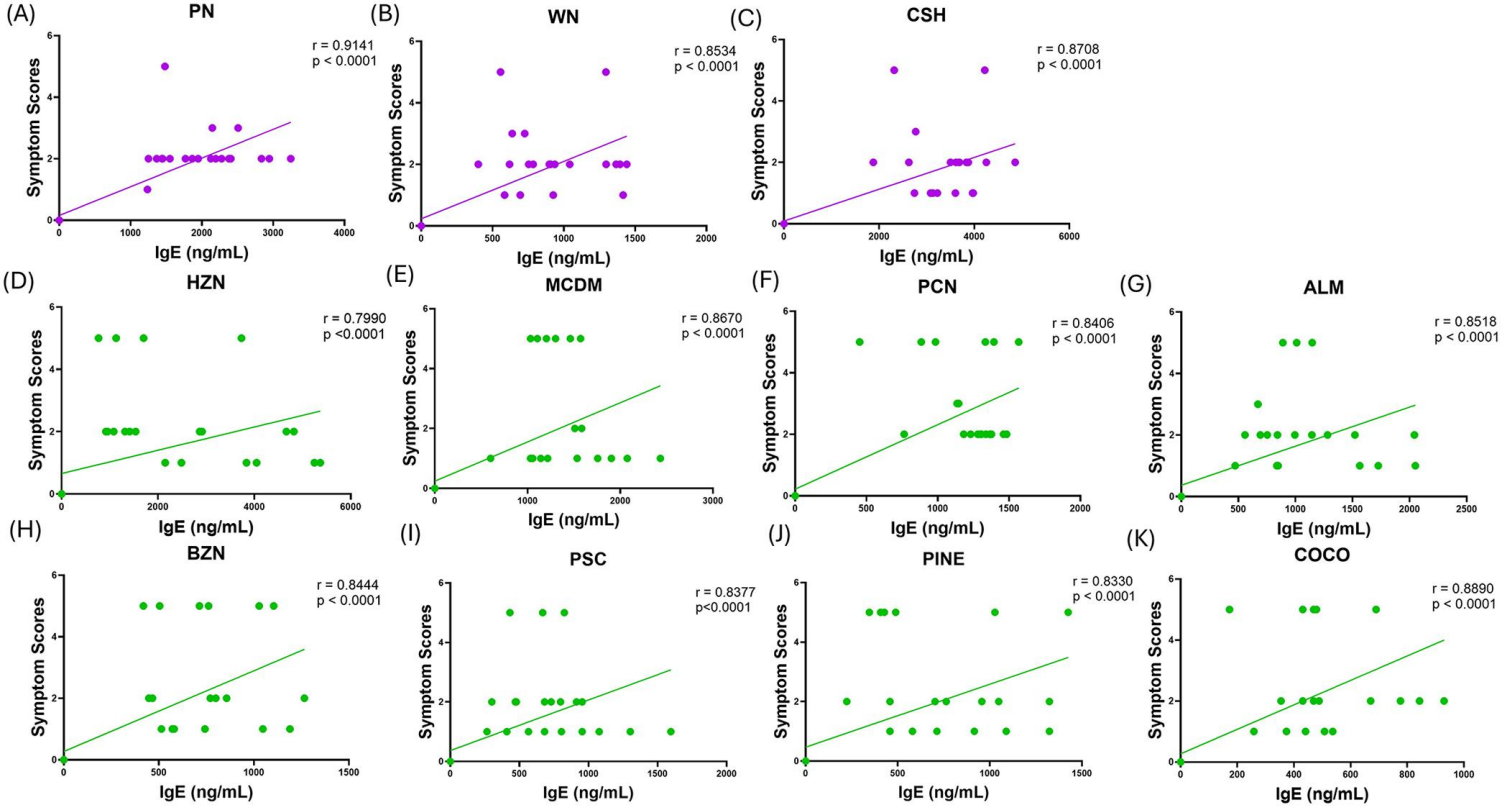
Correlation of IgE levels and anaphylaxis symptom scores of individual primed and unprimed allergens. C3H/HeJ mice (*n* = 24) were sensitized intraperitoneally (i.p.) with 500 μg of peanut, cashew, and walnut (PN/CSH/WN) crude extract adsorbed in 2 mg alum weekly for 3 weeks, then blood was drawn and IgE levels were evaluated. At week 4, mice were challenged over 10–day period and symptom scores were evaluated. Data represents the scores recorded at 40 min post–challenge. Pearson r correlations and simple linear regression analysis were performed in GraphPad for primed allergens **(A–C)** and unprimed allergens **(D–K)**. ALM, almond; BZN, Brazil nut; CSH, cashew; COCO, coconut; HZN, hazelnut; MCDM, macadamia nut; PCN, pecan; PINE, pine nut; PN, peanut; PSC, pistachio; WN, walnut.

Correlation of IgE to anaphylaxis symptom score for each individual nut was conducted. sIgE levels were highly positively correlated with anaphylaxis symptom scores upon challenge for all primed nuts, PN (*r* = 0.9141), WN (*r* = 0.8534), and CSH (*r* = 0.8708) (*p* < 0.0001, Figures 4A–C). IgE levels were strongly positively (SP) correlated with anaphylaxis symptom score upon challenge for the unprimed, cross-reactive nuts, including HZN (*r* = 0.7990, *p* < 0.0001, Figure 4D), MCDM (*r* = 0.8670, *p* < 0.0001, Figure 4E), PCN (*r* = 0.8406, *p* < 0.0001, Figure 4F), ALM (*r* = 0.8518, *p* < 0.0001, Figure 4G), BZN (*r* = 0.8444, *p* < 0.0001, Figure 4H), PSC (*r* = 0.8377, *p* < 0.0001, Figure 4I), PINE (*r* = 0.8330, *p* < 0.0001, Figure 4J), and COCO (*r* = 0.8890, *p* < 0.0001, Figure 4K). This demonstrates that the immunological parameters of primed nuts correlate with the clinical parameters in our model, and this is extended to the unprimed nut allergens, establishing severe clinical outcomes.

### 3.4 Correlation of primed allergens IgE production and anaphylaxis symptom scores

**Figure 5:**
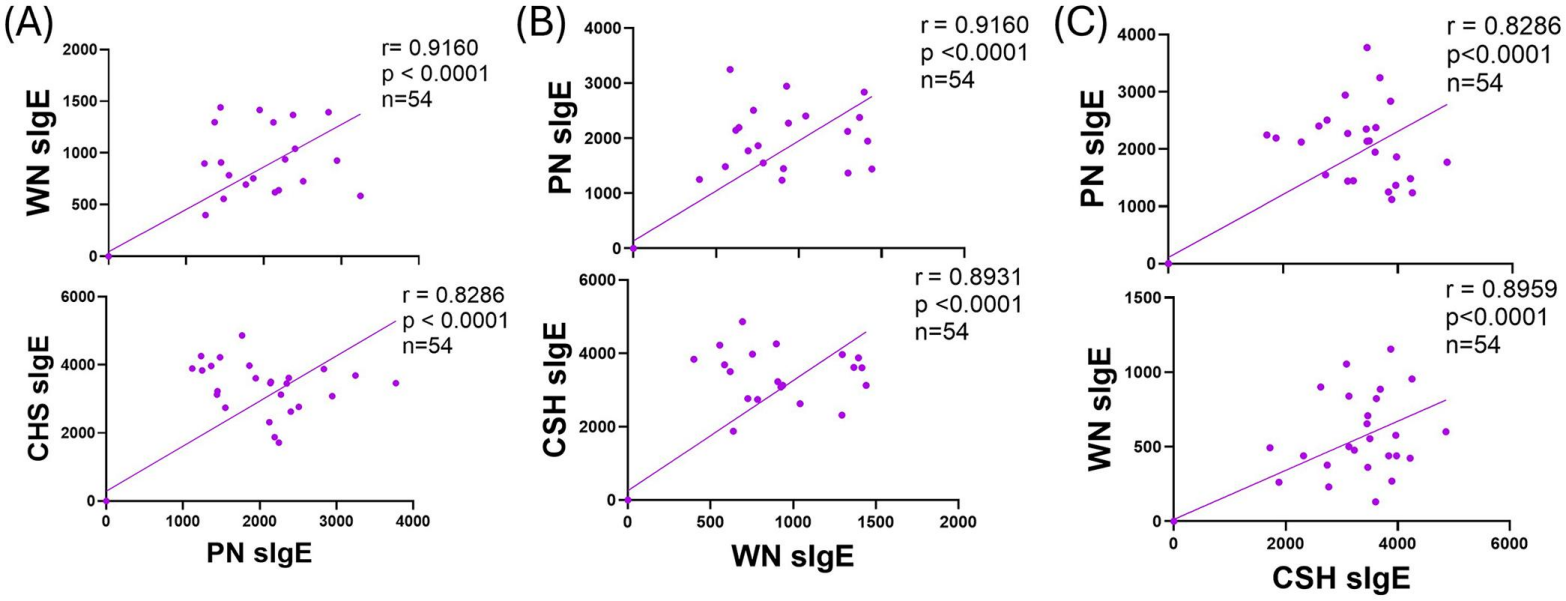
Correlation of primed allergen IgE production. C3H/HeJ mice (*n*= 24) were sensitized intraperitoneally (i.p.) with 500 μg of peanut, cashew, and walnut (PN/CSH/WN) crude extract adsorbed in 2 mg alum weekly for 3 weeks, then blood was drawn and IgE levels were evaluated. Pearson r correlations and simple linear regression analysis were performed in GraphPad comparing to **(A)** PN, **(B)** WN, and **(C)** CSH. CSH, cashew; PN, peanut; WN, walnut.

As PN, CSH, and WN are the most common food allergies and are common in MFA sufferers, we asked in our model whether the IgE between primed nuts were correlated. In this model, PN sIgE very strongly positively (VSP) correlated with WN (*r* = 0.9169) and SP correlation with CSH (*r* = 0.8286) sIgE levels (*p* < 0.0001 Figure 5A). WN sIgE VSP correlated with PN (*r* = 0.9160) and strongly positively correlated CSH (*r* = 0.8913) sIgE levels (*p* < 0.0001 Figure 5B). For CSH sIgE, there was a SP correlation to both PN (*r* = 0.8286) and WN (*r* = 0.8959) sIgE levels (*p* < 0.0001 Figure 5C).

**Figure 6:**
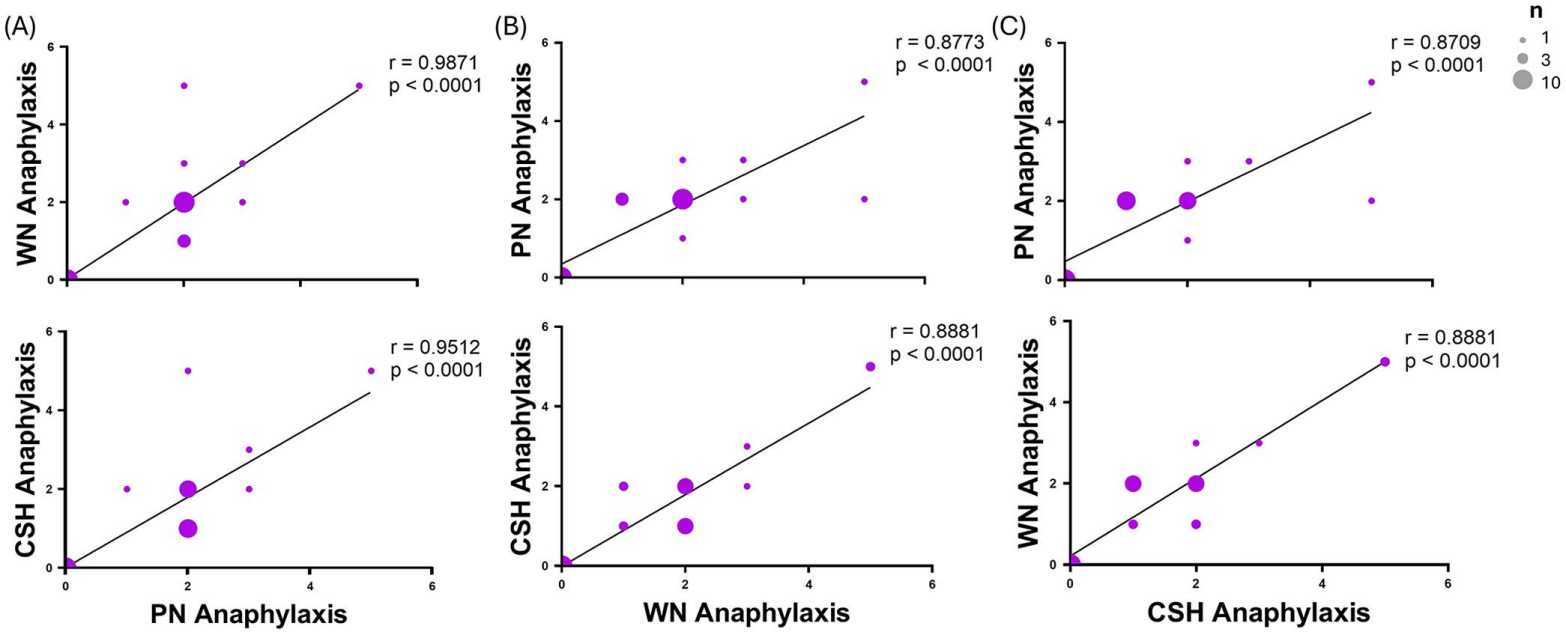
Correlation of primed allergen anaphylaxis symptom scores. C3H/HeJ mice (*n* = 24) were sensitized intraperitoneally (i.p.) with 500 μg of peanut, cashew, and walnut (PN/CSH/WN) crude extract adsorbed in 2 mg alum weekly for 3 weeks. At week 4, mice were challenged over 10-day period and symptom scores were evaluated. Data represents the scores recorded at 40 min post-challenge. The size of dots correlates to the number of samples at a score. Pearson r correlations and simple linear regression analysis were performed in GraphPad comparing to **(A)** PN, **(B)** WN, and **(C)** CSH. CSH, cashew; PN, peanut; WN, walnut.

We next asked whether the anaphylactic symptom scores are correlated between the primed nuts. We saw that with PN there was a VSP correlation with WN (*r* = 0.9871) and CSH (*r* = 0.9512) anaphylaxis symptom scores (*p* < 0.0001, Figure 6A). Further, there was a SP correlation of WN with PN (*r* = 0.8773) and CSH (*r* = 0.8881) anaphylaxis symptom scores (*p* < 0.0001, Figure 6B). Lastly, there was a SP correlation of CSH with PN (*r* = 0.8709) and WN (*r* = 0.8881) anaphylaxis symptom scores (*p* < 0.0001, Figure 6C). This demonstrates that there are a strong sensitization and induction of anaphylaxis that is shared among the primed allergens.

### 3.5 Correlation of primed allergens with cross-reactive tree nut allergens IgE production

**Figure 7:**
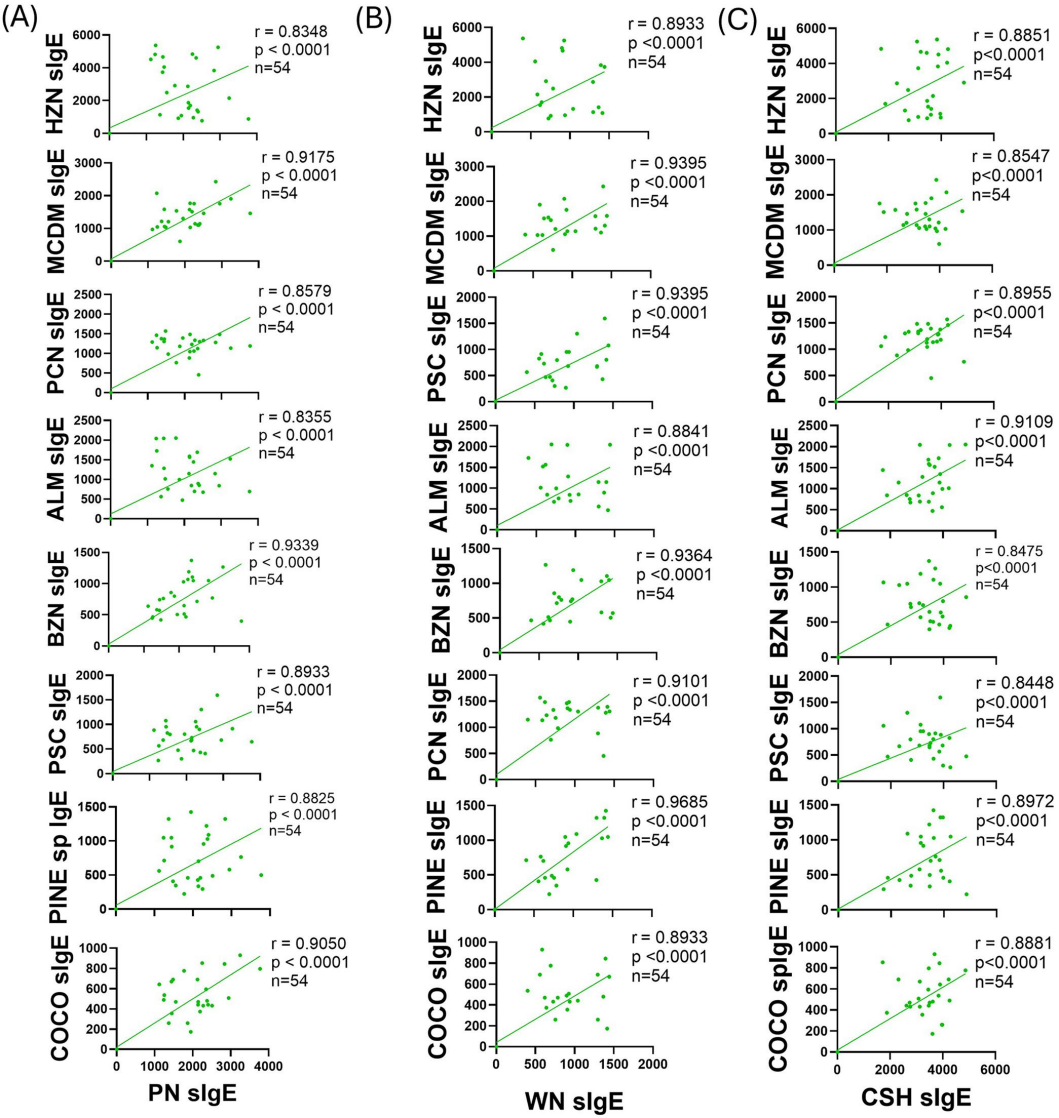
Correlation of primed allergen IgE production with unprimed tree nut allergens IgE production. C3H/HeJ mice (*n* = 24) were sensitized intraperitoneally (i.p.) with 500 μg of peanut, cashew, and walnut (PN/CSH/WN) crude extract adsorbed in 2 mg alum weekly for 3 weeks, then blood was drawn and IgE levels were evaluated. Pearson r correlations and simple linear regression analysis were performed in GraphPad comparing all unprimed nuts to **(A)** PN, **(B)** WN, and **(C)** CSH. ALM, almond; BZN, Brazil nut; CSH, cashew; COCO, coconut; HZN, hazelnut; MCDM, macadamia nut; PCN, pecan; PINE, pine nut; PN, peanut; PSC, pistachio; WN, walnut.

To establish the immunological correlation of the most common food allergies, PN, CSH, and WN, with some of the other tree nuts in multi-food allergic patients, we performed correlation analysis on the expression of cross-reactive IgE levels following sensitization. The levels of PN sIgE levels VSP correlated with BZN (*r* = 0.9339), MCDM (*r* = 0.9175), and COCO (*r* = 0.9050) sIgE levels (*p* < 0.0001, for all; Figure 7A). PN sIgE production SP correlation with PSC (*r* = 0.8933), PINE (*r* = 0.8825), PCN (*r* = 0.8579), ALM (*r* = 0.8355), and HZN (*r* = 0.8348) sIgE production (*p* < 0.0001, for all; Figure 7A). The production of WN sIgE VSP correlated with PINE (*r* = 0.9685), MCDM (*r* = 0.9395), PSC (*r* = 0.9395), BZN (*r* = 0.9364) and PCN (*r* = 0.9101) sIgE production (*p* < 0.0001, for all; Figure 7B). Further, WN levels SP correlated with HZN (*r* = 0.8933), COCO (*r* = 0.8933), and ALM (*r* = 0.8841) sIgE levels (*p* < 0.0001, for all; Figure 7B). CSH sIgE production VSP correlated with ALM sIgE production (*r* = 0.9109, *p* < 0.0001; Figure 7C). CSH sIgE levels were SP correlated with PINE (*r* = 0.8962), PCN (*r* = 0.8955), COCO (*r* = 0.8881), HZN (*r* = 0.8851), MCDM (*r* = 0.8547), PSC (*r* = 0.8448), and BZN (*r* = 0.8475) sIgE levels (*p* < 0.0001, for all; Figure 7C). For all primed allergens, there was no moderate, weak, or negligible correlation of cross-reactive nuts. This confirms that sensitization to WN, PN and CSH produces a meaningful serological cross-reactivity with other TNs.

### 3.6 Correlation of primed allergens with cross-reactive tree nut anaphylaxis symptom scores

**Figure 8:**
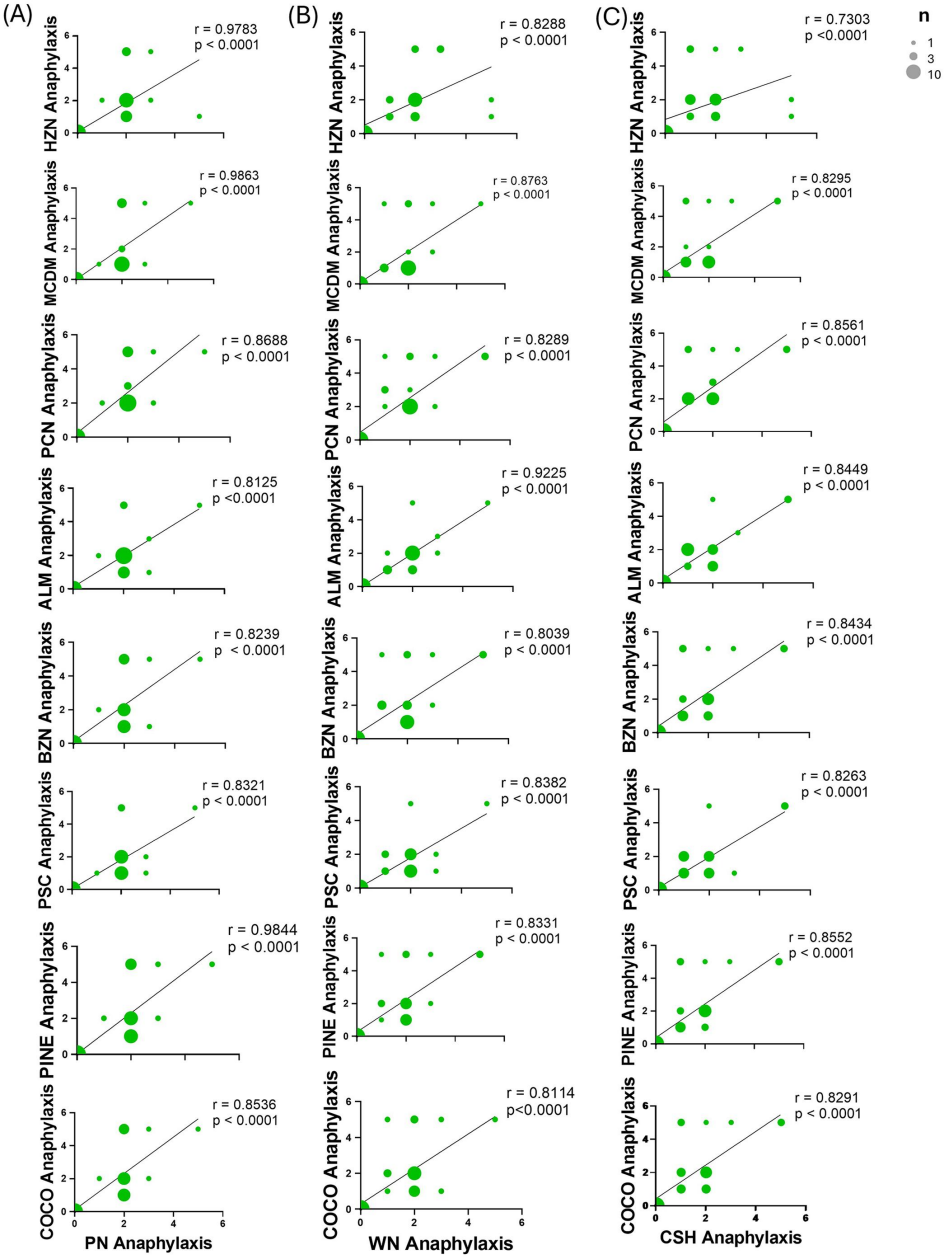
Correlation of primed allergen anaphylaxis symptom scores with unprimed tree nut allergens anaphylaxis symptom scores. C3H/HeJ mice (*n* = 24) were sensitized intraperitoneally (i.p.) with 500 μg of peanut, cashew, and walnut (PN/CSH/WN) crude extract adsorbed in 2 mg alum weekly for 3 weeks. At week 4, mice were challenged over 10-day period and symptom scores were evaluated. Data represents the scores recorded at 40 min post-challenge. The size of dots correlates to the number of samples at a score. Pearson r correlations and simple linear regression analysis were performed in GraphPad comparing all unprimed nuts to **(A)** PN, **(B)** WN, and **(C)** CSH. ALM, almond; BZN, Brazil nut; CSH, cashew; COCO, coconut; HZN, hazelnut; MCDM, macadamia nut; PCN, pecan; PINE, pine nut; PN, peanut; PSC, pistachio; WN, walnut.

To further establish the correlation of clinical outcomes of the most common food allergies, PN, CSH, and WN, with some of the other tree nuts in multi-food allergic patients, we performed correlation analysis on peak anaphylaxis symptom scores induced upon challenge with unprimed nut allergens. The anaphylaxis symptom scores induced with PN were VSP correlated with MCDM (*r* = 0.9863), PINE (*r* = 0.9844), and HZN (*r* = 0.9783) anaphylaxis symptom scores following challenge (*p* < 0.0001, for all; Figure 8A). PN anaphylaxis symptom scores were SP correlated with PCN (*r* = 0.8688), COCO (*r* = 0.8536), PSC (*r* = 0.8321), BZN (*r* = 0.8239), and ALM (*r* = 0.8125) symptom scores (*p* < 0.0001, for all; Figure 8A). The induction of anaphylaxis symptoms with WN challenge were VSP correlated with induction of anaphylaxis symptoms with ALM challenge (*r* = 0.9225, *p* < 0.0001; Figure 8B). WN anaphylaxis symptom scores were SP correlated with MCDM (*r* = 0.8763), PSC (*r* = 0.8382), PINE (*r* = 0.8331), PCN (*r* = 0.8289), HZN (*r* = 0.8288), COCO (*r* = 0.8114), and BZN (0.8039) anaphylaxis symptom scores following challenge (*p* < 0.0001, for all; Figure 8B). The anaphylaxis symptoms induced by CSH were SP correlated with all eight tree nuts, PCN (*r* = 0.85661), PINE (*r* = 0.8552), ALM (*r* = 0.8449), BZN (*r* = 0.8434), MCDM (*r* = 0.8295), COCO (*r* = 0.8291), PSC (*r* = 0.8263), and HZN (*r* = 0.7303), induction of anaphylaxis symptom scores (*p* < 0.0001, for all; Figure 8C). As was seen with IgE levels, for all primed allergens there was no moderate, weak, or negligible correlation of the cross-reactive nuts regarding anaphylaxis symptom scores. This confirms that sensitization to WN, PN and CSH positively correlated with severe reactions to the other tree nuts.

## 4 Discussion

The lack of effective treatments and limited animal models for PN/TN MFA is a critically important and unmet need (28, 34). This study is a novel attempt to elucidate a mechanism for this phenomenon which affects almost 30% of children and 20% of adults with food allergies in the United States. Through our established sensitization and challenge protocols, we were able to assess sIgE levels following sensitization to primed and cross-reactive nuts, and the anaphylaxis symptom scores upon challenge with these primed and secondary nuts. Overall, there was a rise in PN, WN, CSH, and all non-primed TN sIgE levels following sensitization with just PN, WN, and CSH.

Previous work has demonstrated significant increases in IgE and development of anaphylaxis symptom scores compared to naïve mice across a variety of food groups including legumes, TN, shellfish, and fish (28). However, our work focused on specifically the highly prevalent MFA characterized by PN/TN allergies and included nearly all clinically important nuts. Current methods for TN allergy diagnosis include clinical history, sIgE, skin prick testing (SPT), and oral food challenges (OFC). sIgE and SPT assist in determining if OFC is needed, as it is time-consuming and comes with a potentially dangerous risk (7). To combat the limitations of these diagnostic methods, component-resolved diagnostics (CRD) have been developed, which utilizes diagnostically relevant molecular epitopes of recombinant or purified allergens recognized by sIgE (7). Cellular testing, including basophil activation tests (BAT) and mast cell activation tests (MAT), are specific and sensitive options, if accessible (7). Currently, the American Academy of Allergy, Asthma & Immunology (AAAAI) advises individuals with PN allergies to avoid all tree nuts. This can affect nutritional intake and cause large dietary limitations. Further, avoidance is not a reliable solution. Treating TN allergies as one diagnosis is not scientifically sound (35). While the correlation of WN and PCN, CSH and PSC, and ALM and HZN (13, 29), COCO and ALM (36), and ALM and PINE (37) have been demonstrated, differences in TN allergenicity and decision thresholds are underwhelming. It is important to note that subsequent to those studies and the completion of this study, the U.S. Food and Drug Administration no longer classify COCO as a TN, but a drupe, reflecting its distinct botanical origin and lower allergenicity compared to true TNs (38). Although COCO was grouped as a TN in this and prior literature, these new guidelines suggest future studies should consider analyzing COCO separately to avoid overestimation of TNA prevalence and refine clinical recommendations.

In the study conducted by Maloney et al., specific IgE levels between most of the individual tree nuts were moderately correlated. Specifically, the correlation of sIgE levels in patients showed moderate correlations of PN with ALM and weak correlations with PCN, HZN, CSH, PSC, and WN. For CSH, moderate correlations were observed with PSC, HZN, ALM, PCN, with weak correlations with WN and PN sIgE. Lastly, serum WN sIgE levels correlated strongly with PCN, moderately with HZN, and weakly with PSC, CSH, and ALM. However, it is important to note that in this study, clinical reactivity to the food was not confirmed. Other studies have confirmed that while elevated IgE levels are associated with an increased risk for allergic reactions, there has not been demonstration of a consistency in the prediction of anaphylaxis severity in PNA and TNA individuals (39, 40). This is explained by factors including the presence of specific allergenic proteins, the immune response of the individual, and coexisting atopic conditions.

In this model, a strong cross-IgE sensitization to the eight tree nuts is evident with relevant clinical outcome, despite not being exposed previously. Taken together, the strongly positive correlations of sIgE production that also resulted in the development of anaphylactic reactions with strongly positive correlations highlights cross reactivity between PN and MCDM and PINE; WN and MCDM, ALM, and PINE; and CSH and ALM, PCN, and PINE (*r* > 0.83, *p* < 0.0001 for all). We have demonstrated that with a PN allergy, there is a strong positive correlation with a rise in PINE and MCDM IgE levels, and hence it would suggest an ability to mount an immune response to MCDM when a PN allergy exists. This was confirmed with the strong positive correlation with clinical reactivity, evidence by anaphylactic reactions following challenge. Cross-reactivity between PN and PINE has been previously suggested (41). This is the first study to emphasize the correlation between PN and MCDM. Veiga et al. suggest that no cross-reactivity of MCDM to PN was detected, however this was done with a SPT in one patient and therefore a larger sample size is warranted. Further, SPT correlates with a likelihood of positive challenge however does not correlate with disease severity and evidence suggests SPT technique affects outcomes and skin reactivity may vary with age (42). Therefore, our assessment of correlation upon challenge *in vivo* more closely demonstrates the gold standard oral food challenge that is the most reliable for human patients with PN and TN allergies (43). We have also demonstrated for the first time a strong positive correlation of sIgE levels between WN and PINE, and WN and ALM. The observed WN and MCDM cross-reactivity has been previously described, highlighting MCDM containing WN cross-reactive IgE binding proteins (44). In a Spanish cohort, Gutierrez-Diaz et al. observed that WN extract was not cross-reactive with MCDM (45). However, even authors of this study note the small sample size and regional differences due to dietary habits and pollen exposure that accounts for differences seen even compared to Australian and Japanese population studies. These inherent differences can be accounted for in this animal model system. Further this is the first study to demonstrate the correlation of CSH and PCN, and CSH and PINE. CSH has been previously grouped with ALM as moderately cross-reactive along with HZN, BZN, and PSC (46). The benefit of our study is that the correlation of sIgE levels agreed with what was observed upon food challenge anaphylaxis symptom scores.

Infant feeding guidelines recommend introduction commonly allergenic foods, including peanuts, between 4 and 6 months of age, a period of time thought to be a developmental window whereby exposure may promote oral tolerance and reduce the risk of FA (47). Introducing less allergenic foods in infants is also recommended to reduce the risk of developing food allergies (48). Lower-correlated TNs can also act similarly to food allergy oral immunotherapy, enabling tolerance and decreasing likelihood for an allergic reaction through reduced allergenicity. HZN, for example, can serve as part of an early introduction regimen over total avoidance in the context of CSH allergy, as we would expect to see a less severe reaction that may provide protection against others. Our model can also serve as a useful tool to test these possibilities. Our model can also be utilized to assess the efficacy of prophylactic or therapeutic interventions for multi-food allergies. The assessment of food-specific sensitization including serological levels of IgE, IgG1, IgG2a, and the B and T cell responses, along with the determination of cross-reactivity and functional clinical reactivity of anaphylaxis upon oral food challenge can be achieved with this model. Treatments given prophylactically during or before sensitization can be tested by assessing IgE levels. Treatment efficacy following sensitization can be determined by reductions in IgE levels, prevention of cross-reactivity and other clinical reactions. Both models can be supplemented by measuring circulating mediators of allergic reactions, including histamine and mast cell proteases, assessing basophil/mast cell activation *ex vivo*, and elucidating the T and B cell responses.

There are various mouse models for *in vivo* food allergy studies, serving differential research purposes. BALB/c mice are one example, due to their Th2-skewed immune responses enabling the development of IgE-mediated reactions in response to allergens. A limitation of BALB/c mice includes the lack of severe anaphylaxis symptoms, making them less ideal for severe allergy research. Mice lacking the high-affinity IgE receptor (FcεR1-deficient) are used to study the roles of IgE and FcER1 signaling in food allergies. A limitation of these mice includes the inability to use in investigating spontaneous allergic symptoms. Ovalbumin (OVA)-sensitized mice as a model allergen in food allergy is useful for basic immunological responses and tolerance mechanisms. A limitation of these mice is that they do not fully mimic human food allergens, so the clinical application is limited. Lastly, C3H/HeJ mice, chosen for this study, are susceptible to anaphylaxis reactions upon exposure to food allergens with significant IgE responses. C3H/HeJ mice can stimulate cross-reactivity and allergy to multiple foods and hence can be used for development of potential treatments.

Limitations include our use of a combination of PN, WN and CSH and not using single allergens for sensitization, which was chosen to mimic the mutli-food allergic population clinical reality and interactions of different foods. Future work would benefit from single nut sensitization to demonstrate true cross-reactivity and effects of epitope spreading and competition. Sequential sensitization controls can also be used to test the effects of priming vs. co-sensitization. Correlation analysis and pre-defined statistical methods allowed us to maintain biologically meaningful biological cross-reactivity data. We were able to provide analysis that is not captured *in vitro* or from a serological panel, including predisposition to clinically meaningful reactions to less common tree nut allergens. Assessment of shared T cell reactivity can also supplement the correlations between cross-reactive IgE and severity of anaphylaxis symptom scores to validate this role of IgE. Computational modeling and immunoinformatic tools can be used to identify structural similarities between cross-reactive nuts and study binding affinities between shared cross-reactive peptides. Characterization of T cell and B cell cross-reactive epitopes could improve the understanding of cross-reactivity mechanisms and design diagnostic and prognostic assays to predict cross-reactivity to an initiator allergen source. Other mechanisms to assess include IgG isotypes and the mast cell and basophil activation readouts or mediator release in this cross-reactivity model. Both oral and systemic sensitization, followed by oral challenges, have been used previously to generate food allergy models, with systemic being the most efficient (49–51). Alum was used as an adjuvant for sensitization in this study, as adjuvants are necessary for immunological studies as they serve to enhance the immune response, enabling a detectable reaction in food allergy and asthma models (52). Food hyper-sensitivity models utilize this potent adjuvant as it breaks down the innate tolerance and promotes strong Th2 polarization in the presence of food protein (53). This reaction may be more severe than natural human responses and may not perfectly replicate a human's allergic response without adjuvant. This could lead to more pronounced IgE levels and symptom scores than a model that was not administered the adjuvant, however this study resulted in meaningful clinical results utilizing this method. Human data to corroborate including serological panels and clinical datasets would enhance the translatability of this work. Therefore, careful consideration and interpretation must be taken for translatability to human allergic responses at this time. Future work may benefit from humanized mouse models to address this limitation.

Our findings demonstrate that systemic sensitization of mice to peanut and common tree nuts, walnut and cashew, elicited antigen-specific IgE responses that cross-reacted with unprimed tree nut allergens. Importantly, this serological cross-reactivity was accompanied by functional evidence of clinical reactivity, as oral challenge induced anaphylaxis *in vivo*. Correlation analyses between anaphylaxis and IgE reactivity further helped delineate the statistical associations underlying individual cross-sensitization to each primed nut, strengthening the interpretation of specific allergen relationships. Collectively, these results highlight that multi-allergen sensitization can extend beyond the initially targeted allergens and may drive clinically relevant reactivity patterns. However, the reliance on murine systemic sensitization models necessitates caution in extrapolating directly to human allergy, whereby sensitization typically occurs through mucosal routes and involves complex environmental and genetic factors. This model, however, can appropriately assess modulation of disease-specific mechanisms by a therapeutic intervention, including IgE production, effector cell activation, and tolerance induction, which are conserved across species. Further studies integrating human immunological and clinical data will be essential to clarify the true translational significance of the proposed cross-reactivity. Nonetheless, the observation that sensitization to a single nut may extend risk to multiple tree nuts underscores the need for careful clinical consideration and highlights this model's value for future therapeutic development.

## Data Availability

The original contributions presented in the study are included in the article/Supplementary Material, further inquiries can be directed to the corresponding author.
